# Psychometric Validation of Trust, Commitment, and Satisfaction Scales to Measure Marital Relationship Quality Among Newly Married Women in Nepal

**DOI:** 10.3390/ijerph22091457

**Published:** 2025-09-20

**Authors:** Lakshmi Gopalakrishnan, Nadia Diamond-Smith, Hannah H. Leslie

**Affiliations:** 1 Institute for Global Health Sciences, University of California, San Francisco, 550 16th St., Floor 3, San Francisco, CA 94143, USA; nadia.diamond-smith@ucsf.edu; 2 Department of Epidemiology and Biostatistics, University of California, San Francisco, 550 16th St., Floor 3, San Francisco, CA 94143, USA; 3 Division of Prevention Science, Department of Medicine, University of California, San Francisco, 550 16th St., Floor 3, San Francisco, CA 94143, USA; hannah.leslie@ucsf.edu

**Keywords:** marital relationship quality, marital quality, psychometric validation, scale development, marriage and health, newly married couples, Nepal, South Asia

## Abstract

Marital relationship quality significantly influences health outcomes, but validated measurement tools for South Asian populations remain limited. To validate scales measuring trust, commitment, and satisfaction as key components of marital relationship quality among newly married women in Nepal, we conducted a two-wave psychometric validation study in rural Nawalparasi district. The study included 200 newly married women aged 18–25 years, with 192 participants (96% retention) completing 6-month follow-up. We assessed factor structure, internal consistency, test-retest reliability, and criterion validity of trust (eight items), commitment (five items), and satisfaction (seven items) scales using exploratory and confirmatory factor analysis. Exploratory factor analysis identified single-factor solutions for trust and commitment scales and a two-factor model for satisfaction. Confirmatory factor analysis confirmed these structures, with satisfaction comprising marital conflict/dissatisfaction (four items) and general satisfaction (two items) subscales. All scales demonstrated good internal consistency (Cronbach’s α: 0.79–0.96) and significant criterion validity correlations with relationship happiness (r = 0.63–0.72, *p* < 0.001). Test-retest reliability showed moderate to low stability (r = 0.21–0.51), likely reflecting genuine relationship changes in early marriage. The validated scales provide reliable tools for assessing relationship quality in South Asian contexts, enabling research on marriage-health associations and evidence-based interventions.

## 1. Introduction

Marriage in Nepal, a near-universal and culturally significant institution, is undergoing rapid changes [1,2]. Historically arranged by parents or relatives, there has been a recent shift towards self-choice “love marriages”, accompanied by increases in marriage age and divorce rates, though these remain low compared to global standards [3,4,5,6,7,8,9]. These evolving dynamics underscore the critical importance of understanding marital relationships and their dynamics and quality in contemporary Nepali society.

Marital relationship quality profoundly influences physical and mental well-being beyond the marital unit itself [10,11]. Meta-analytic evidence from over 72,000 individuals demonstrates significant associations with health outcomes, including reduced mortality, better cardiovascular health, enhanced personal well-being, and reduced anxiety symptoms, with effect sizes comparable to established health behaviors like diet and exercise [11,12,13]. However, there is a notable research gap in non-Western settings, particularly Nepal. While existing Nepali studies have examined determinants of marital quality [14] and its association with women’s agency [15] and mental health [16], they highlight the need for more comprehensive understanding and validated measurement tools in this cultural context.

### 1.1. Theoretical Framework for Marital Relationship Quality

Marital relationship quality has evolved from unidimensional concepts [17,18] towards more multidimensional constructs capturing distinct but interrelated relationship aspects [19]. Yet, a significant challenge in this field is the lack of consensus on what constitutes a “good” marital relationship and how to measure it consistently across different cultural contexts [20]. We use three contemporary relationship theories that converge on several core dimensions as fundamental to relationship quality assessment. Social Exchange Theory says that relationship quality depends on the balance of rewards and costs, with trust serving as a crucial mechanism for reducing relationship uncertainty and enabling cooperative behavior, while commitment represents investment in future relationship rewards [21,22]. Attachment Theory highlights trust as the cornerstone of secure emotional bonding, claiming that early attachment experiences influence how adults perceive a partner’s dependability and capacity for emotional connection [23,24]. Finally, Sternberg’s Triangular Theory of Love positions commitment as one of three core components of enduring love relationships, alongside passion and intimacy. Commitment reflects a conscious, reasoned choice to stay invested in a relationship over time [25,26]. Cross-cultural validation studies in diverse settings have consistently identified trust, commitment, and satisfaction as core relationship quality indicators, though their relative importance and expression may vary across cultural contexts [27,28].

Our study focuses on trust, commitment, and satisfaction as theoretically grounded core dimensions that capture both cognitive and affective relationship aspects while demonstrating cross-cultural relevance in non-Western populations.

### 1.2. Trust as a Fundamental Relationship Component

Mutual trust is crucial for establishing close relationship [29,30,31,32], enabling couples to experience shared joy and empathy during hardships, leading to deeper understanding and genuine intimacy [32,33,34]. Trust plays a central role in marital relationships by easing doubts about a partner’s intentions, fostering the emotional safety needed for open self-disclosure, and supporting collaboration even when goals diverge [35,36]. From a developmental perspective, trust builds through ongoing positive exchanges and reliable partner responsiveness, forming a secure base that helps couples navigate conflict and adversity [36,37]. Conversely, absence of trust results in anxiety, insecurity, and relationship breakdown [37]. When trust is violated or absent, couples experience increased vigilance, emotional distance, and difficulty engaging in the vulnerable behaviors necessary for intimacy [31,36]. Trust violations, including broken confidence, inconsistent behavior, and emotional neglect, are primary predictors of relationship dissolution and ongoing relationship distress [33,37].

Cross-cultural research reveals that while trust appears universally important for relationship quality, its expression and development may vary significantly across cultural contexts. In collectivist cultures like Nepal, trust may extend beyond the dyadic relationship to include family networks and community expectations, potentially affecting both its measurement and cultural meaning. While Western measures like the 8-item Dyadic Trust Scale [38] exist, but their applicability in the Nepali context remains unexplored. This measurement gap highlights the critical need for testing the applicability of trust scales as understood and experienced within South Asian marital relationships. 

### 1.3. Commitment as a Relationship Stability Indicator

Commitment, one of Sternberg’s triangular theory of love alongside intimacy and passion, represents the cognitive decision to maintain a long-term relationship [26]. This dimension the cognitive aspect of love and refers to the decision to maintain a long-term relationship with a partner. Commitment reflects both a deliberate intention to maintain the relationship and a deep emotional bond that holds partners together through periods of conflict and changing levels of satisfaction [39]. Rusbult’s Investment Model presents a comprehensive approach to understanding relationship commitment, proposing that it is shaped by three main factors: satisfaction with the relationship, the perceived quality of available alternatives, and the magnitude of investments made—whether emotional, temporal, or financial [22,40].

In arranged marriage contexts common in Nepal, the balance between different commitment types may differ substantially from patterns observed in Western love marriages. Moral and structural commitments often play a more prominent role in the early stages, with personal commitment emerging over time through shared experiences and the gradual development of emotional bonds [41]. Commitment functions as a relationship maintenance mechanism, fostering pro-relationship behaviors such as willingness to sacrifice personal interests for relationship benefit, constructive conflict resolution approaches, and sustained investment of time and effort in relationship improvement [42,43]. Harvey et al. adapted Stenberg’s Triangular Theory of Love into a 5-item scale, with higher commitment levels associated with relationship stability [44,45], relationship maintenance behaviors [46,47], and individual mental and physical outcomes [48]. However, the psychometric properties of Western-developed commitment measures in South Asian populations remain largely unexamined, representing a significant gap for relationship research in these cultural contexts.

### 1.4. Satisfaction as a Relationship Quality Measure

Relationship satisfaction represents individuals’ subjective evaluation of their relationship based on the degree to which it meets their needs, expectations, and desires [49]. Satisfaction demonstrates considerable temporal variability, varying in response to ongoing relationship interactions, life stressors, and changing circumstances [50]. This dynamic nature makes satisfaction both a valuable outcome indicator and a potential predictor of future relationship development and stability.

Theoretical models of relationship satisfaction highlight both comparison processes and need-fulfillment mechanisms. Social comparison perspectives, grounded in social exchange theory, propose that satisfaction is shaped in part by how individuals evaluate their relationship against perceived alternatives, prior relationships, and prevailing social standards for relationship quality [21]. Need fulfillment theories focus on whether relationships satisfy fundamental psychological needs including autonomy, competence, and relatedness, with unmet needs contributing to relationship dissatisfaction [51]. Recent theoretical developments have challenged traditional unidimensional conceptualizations of satisfaction, proposing instead that individuals can simultaneously experience positive and negative feelings about their relationships [52,53]. This bidimensional perspective views satisfaction as comprising both positive elements (intimacy, companionship, emotional support) and negative aspects (conflict frequency, disappointment, relationship distress). These dimensions may operate independently and show different patterns of association with relationship outcomes.

In South Asian contexts, satisfaction may be influenced by distinct cultural factors including fulfillment of traditional gender role expectations, family and community approval of the marriage, adherence to relationship norms, and successful navigation of extended family relationships [14,28]. The collectivist cultural framework may emphasize relational harmony and family integration as key components of satisfaction beyond individual fulfillment.

These three constructs—trust, commitment, and satisfaction, operate synergistically to influence overall relationship quality while maintaining sufficient conceptual distinctiveness to warrant separate measurement and assessment [54]. The theoretical integration reflects a dynamic interplay: trust facilitates the emotional vulnerability and openness necessary for relationship satisfaction, while satisfaction experiences motivate continued investment and deeper commitment to the partnership [55]. Commitment, in turn, promotes trust-building behaviors, patience during periods of temporary dissatisfaction, and willingness to work through relationship challenges rather than seeking alternatives. Empirical research supports the discriminant validity of these constructs while confirming their theoretical interconnections. Meta-analytic evidence demonstrates moderate correlations between trust, commitment, and satisfaction (typically r = 0.40–0.70), indicating related but sufficiently distinct constructs that contribute unique variance to overall relationship quality assessment [56,57]. Factor analytic studies across diverse populations consistently identify these as separate factors in relationship quality models, supporting their theoretical distinctiveness while confirming their practical utility for relationship assessment [58].

While relationship quality measures have been validated in diverse non-Western settings [14,27,59,60], psychometric validation of Western-developed scales in South Asian populations remains limited. This study aims to validate three theoretically grounded dimensions of marital relationship quality in Nepal: trust, commitment, and satisfaction. By establishing psychometrically sound measures of these core constructs, we seek to provide researchers with reliable tools for investigating how marital relationships influence health outcomes in South Asian contexts. By establishing the psychometric properties of these core constructs among newly married women, we seek to provide researchers and clinicians with reliable tools for both investigating how marital relationships influence health outcomes and identifying couples at risk for relationship distress in South Asian contexts. These validated measures will enable research on maternal mental health, family health behaviors, and couple-based health interventions, while contributing to the broader global health research infrastructure for relationship studies in low- and middle-income countries.

## 2. Methods

### 2.1. Study Design and Recruitment

This psychometric validation study utilized data from a longitudinal cohort of 200 newly married women conducted between 2018 and 2020 in Nawalparasi district, Nepal. Following established scale validation methodology, exploratory factor analysis (EFA) was performed on baseline data (n = 200) in February 2018 and confirmatory factor analysis (CFA) on 6-month follow-up data (n = 192). The 6-month interval was selected as optimal for test-retest reliability assessment, balancing measurement stability evaluation with genuine relationship changes that occur as newly married couples adjust to married life. The Terai region is predominantly inhabited by the Madeshi (Hindu) and Muslim communities and shares cultural affinities with neighboring Indian districts. Around 97% of Nepal’s Muslim population resides in the Terai, sharing socio-cultural ties with populations in Uttar Pradesh and Bihar, India. The study population is representative of the Terai region of Nepal.

At baseline, eligibility criteria included: marriage within four months preceding the baseline survey, age 18–25 years, and co-residence with mother-in-law in the same household. A systematic sampling approach was employed: villages were purposively selected within Nawalparasi district, followed by comprehensive household listing to identify all newly married women meeting eligibility criteria. This approach ensured population-based recruitment representative of newly married women in the Terai region. Of 200 women enrolled at baseline, 192 (96% retention rate) completed the 6-month follow-up assessment. Attrition analysis revealed no significant differences in baseline sociodemographic characteristics between completers and non-completers, confirming that the CFA sample remained representative of the original cohort. More details of the broader study sampling design are found elsewhere [61]. This study was conducted according to the guidelines laid down in the Declaration of Helsinki, and all procedures were approved by the Nepal Health Research Council of Nepal and Institutional Review Board of the University of California, San Francisco. Written informed consent was obtained from all participants.

### 2.2. Study Measures

Independent variables (marital relationship quality domains).

Three domains of relationship quality were assessed, as reported by women. These core dimensions were selected based on: (1) their theoretical grounding in established relationship frameworks, (2) their documented associations with health outcomes in Western populations, (3) their emergence in cross-cultural relationship research, and (4) evidence from previous adaptation work in Ethiopia suggesting potential cultural relevance in South Asian contexts [27].

(i) Commitment Scale: This 5-item scale, adapted from the Sternberg Triangular Love Scale [26] by Harvey and colleagues [44], assessed participants’ long-term commitment to their spouse. Items included statements such as, “I can’t imagine ending my relationship with my current husband” rated on a 4-point scale (0 = not at all, 1 = somewhat, 2 = quite, 3 = extremely). Higher scores indicate greater spousal commitment.

(ii) Trust Scale: This 8-item scale, adapted from the Dyadic Trust Scale by Larzelere and Huston [38], assesses trust within the marital relationship. Items included statements such as, “My husband is primarily interested in his own welfare”, using a Likert scale ranging from strongly agree to strongly disagree. Negatively worded items were reverse coded, with higher scores indicating greater marital trust.

(iii) Satisfaction Scale: This 7-item scale, derived from the Dyadic Satisfaction Scale subscale of the Dyadic Adjustment Scale [49], measured relationship satisfaction using four-point frequency ratings. Items such as “How often do you or your husband leave the house after a fight?” were rated on a 4-point scale (0 = all of the time to 3 = never). Negatively worded items were reverse coded, with higher scores indicating greater relationship satisfaction.

Criterion validity measure: Convergent validity was assessed using a single-item global relationship happiness measure: “How happy are you with your relationship overall?” rated on a 4-point scale (1 = very unhappy to 4 = perfectly happy). We hypothesized positive correlations between this measure and all three relationship quality scales.

### 2.3. Psychometric Analyses

The sample characteristics were summarized using means, standard deviations (SD), frequencies, and percentages. A comprehensive psychometric evaluation was conducted using a two-waves of data collected from the same women. Inter-item correlations were examined for each scale prior to factor analysis. Data suitability for factor analysis was assessed using the Kaiser-Meyer-Olkin (KMO) measure of sampling adequacy and Bartlett’s test of sphericity. KMO values ≥0.60 and significant Bartlett’s tests (*p* < 0.05) were considered acceptable for factor analysis. 

Exploratory Factor Analysis: We conducted EFA on baseline data (n = 200) using Stata 18 [62] to determine the optimal factor structure for each scale. We employed a multi-step approach. First, we conducted parallel analysis, which compares the eigenvalues of the observed data with those from randomly generated data [63,64]. Next, we examined the scree plot, visually inspecting for the ‘elbow’ point where the curve levels off, indicating diminishing returns from additional factors [65,66]. We also considered Kaiser’s criterion, which suggests retaining factors with eigenvalues greater than 1.0 [67,68]. When methods yielded different results, we prioritized the parallel analysis as it is generally considered more accurate [69]. Oblique rotation was used given theoretical correlations between relationship quality domains [70,71,72]. Factor loadings ≥0.4 were considered acceptable for item retention [64,73]. Scale scores were computed as unweighted sum scores of all retained items, consistent with standard practice in psychometric validation studies [64]. This approach was chosen over factor-weighted scoring to maintain practical utility and ease of interpretation for future users.

Confirmatory Factor Analysis: CFA was performed on 6-month follow-up data (n = 192) in R Studio 2024.04.1+748 [74] to test EFA-identified factor structures. Model fit was evaluated using: root mean square error of approximation (RMSEA), Tucker–Lewis index (TLI), comparative fit index (CFI), and standardized root mean squared residual (SRMR). Models were considered acceptable if any two of the following criteria were met: RMSEA ≤ 0.06, TLI and CFI ≥ 0.95, SRMR ≤ 0.1 [75,76]. Scale scores for confirmatory factor analysis were computed as unweighted sum scores of retained items. The commitment scale score was the sum of 5 items, the trust scale was the sum of 8 items, and the satisfaction scale yielded two subscale scores: marital conflict/dissatisfaction (sum of 4 items) and general satisfaction (sum of 2 items).

Reliability and Validity Assessment: Internal consistency was assessed using Cronbach’s alpha (α ≥ 0.70 considered acceptable) [77]. Test-retest reliability was evaluated using Pearson correlations between baseline and 6-month scores. Criterion validity was examined through correlations between scale scores and single-item relationship happiness [64,78]. Inter-scale correlations were assessed to evaluate construct validity [79].

Sample Size Justification: Sample size of 200 provided adequate power for factor analysis (>10:1 participant-to-item ratio). This manuscript was prepared following the Strengthening the Reporting of Observational Studies in Epidemiology (STROBE) reporting guideline [80] and checklist [81] to ensure comprehensive reporting of study methodology and results.

## 3. Results

### 3.1. Descriptive Statistics

Table 1 presents characteristics of the 200 newly married women aged 18–25 who contributed to the EFA. Participants had a mean age of 20.4 years (SD = 1.9) with an average of 10 years of education. Most were Hindu (86%), with the remainder identifying as Muslim, Christian, or Buddhist (14%). Ethnically, 53% belonged to indigenous groups, 25% to Dalits/religious minorities, and 23% to Brahmin/Chhetri groups. The majority (70.5%) had arranged marriages, while 29.5% chose their partners. Twenty-six percent engaged in paid work outside the home. Husbands averaged 23.9 years (SD = 2.9), with most completing grades 6–12 (74%) or higher education (15%); only three had no formal schooling.

Table 2 presents descriptive statistics for all scale items at baseline. Commitment scale scores ranged from 0–15 (M = 13.52, SD = 2.77), with individual items averaging 2.65–2.74 on the 4-point scale, indicating generally high commitment levels across participants. Trust scale total scores ranged from 0–24 (M = 18.10, SD = 4.14), with positive trust items averaging 2.32–2.39 and reverse-coded items showing lower means (0.78–1.00), suggesting overall high trust levels. Satisfaction scale scores ranged from 3–21 (M = 18.50, SD = 2.65), with most items showing high satisfaction levels, particularly low frequencies of divorce consideration (M = 2.95) and leaving after fights (M = 2.99).

Inter-item correlations were examined to assess item quality and potential range restriction. For the commitment scale, correlations ranged from r = 0.59 to r = 0.85 (M = 0.72), indicating strong relationships among all items. Trust scale inter-item correlations ranged from r = 0.32 to r = 0.87 (M = 0.55). Satisfaction scale correlations ranged from r = 0.26 to r = 0.72 (M = 0.42), displaying the greatest variability with four correlations below 0.30, primarily between conflict-related items and positive relationship indicators. This heterogeneous correlation pattern supported the subsequent identification of a two-factor structure for the satisfaction scale. The weakest correlation (r = 0.26) was observed between items measuring conflict frequency.

Factor analysis assumptions were met for all scales. KMO values demonstrated good to excellent sampling adequacy: commitment scale (KMO = 0.818), trust scale (KMO = 0.899), and satisfaction scale (KMO = 0.797). Bartlett’s tests of sphericity were significant for all scales (all *p* < 0.001), confirming adequate intercorrelations for factor extraction.

### 3.2. Psychometric Properties of the Scales

Commitment scale: EFA on baseline data (n = 200) supported a unidimensional structure for the commitment scale. Parallel analysis indicated one factor with eigenvalues exceeding random data expectations, confirmed by scree plot examination showing a clear drop-off after the first factor. The single factor (eigenvalue = 3.6) accounted for 99% of cumulative variance, with all five items loading above 0.4 (range: 0.76–0.90), supporting a robust one-factor solution.

Trust Scale: The trust scale similarly demonstrated a unidimensional structure. Parallel analysis and scree plot inspection both supported a one-factor solution. The single factor (eigenvalue = 4.5) explained 98% of cumulative variance, with all eight items meeting the 0.4 loading threshold (range: 0.52–0.90), confirming a unidimensional trust construct.

Satisfaction Scale: EFA revealed a two-factor structure for the satisfaction scale. Parallel analysis confirmed both factors exceeded random data expectations, supported by scree plot examination. The two-factor solution yielded:

- Factor 1: Marital conflict/dissatisfaction (4 items, eigenvalue = 3.1, factor loadings: 0.74–0.81) included items about divorce consideration, leaving after fights, marital regret, and quarreling frequency.
- Factor 2: General satisfaction (2 items, eigenvalue = 0.83, factor loadings: 0.78–0.82) included items about relationship wellbeing and confiding behaviors.
- Factor loadings for all scales are presented in Table 3.

Based on these factor structures, we recommend that future users compute unweighted sum scores: total all retained items for the commitment and trust scales and compute separate subscale scores for the satisfaction scale’s two factors (marital conflict/dissatisfaction: 4 items; general satisfaction: 2 items).

### 3.3. Confirmatory Factor Analysis (CFA)

CFA was conducted on 6-month follow-up data (n = 192) to test the factor structures identified in EFA. CFA tested the 6-item, two-factor satisfaction model identified in EFA, excluding the dropped item. Table 4 presents standardized factor loadings and Table 5 shows model fit statistics.

CFA results confirmed the factor structures identified in EFA. The commitment scale single-factor model demonstrated acceptable fit, with CFI (0.983), TLI (0.966), and SRMR (0.012) meeting criteria, though RMSEA (0.150) exceeded the preferred threshold. Similarly, the trust scale single-factor model showed acceptable fit based on CFI (0.954), TLI (0.936), and SRMR (0.028), despite elevated RMSEA (0.149). For the satisfaction scale, CFA tested both one-factor and two-factor models to confirm the EFA-derived structure. The two-factor model demonstrated superior fit (RMSEA = 0.088, CFI = 0.981, TLI = 0.965, SRMR = 0.035) compared to the one-factor model, which showed poor fit across all indices (RMSEA = 0.257, CFI = 0.794, TLI = 0.691, SRMR = 0.084).

Based on our acceptance criteria (at least two of: RMSEA ≤ 0.08, CFI and TLI ≥ 0.95, SRMR ≤ 0.10), all final models demonstrated acceptable fit. The elevated RMSEA values for commitment and trust scales, while above ideal thresholds, are not uncommon in small-scale validation studies and do not preclude scale utility given the strong performance on other fit indices.

Scale scores for CFA were computed as unweighted sum scores of retained items, consistent with the EFA approach. The commitment scale score was the sum of 5 items, the trust scale was the sum of 8 items, and the satisfaction scale yielded two subscale scores: marital conflict/dissatisfaction (sum of 4 items) and general satisfaction (sum of 2 items).

Inter-scale correlations demonstrated moderate to strong associations (r = 0.517–0.720), indicating that the constructs are theoretically related while remaining sufficiently distinct to measure different aspects of marital relationship quality (Table 6).

The correlation pattern supports the validity of the scales. The strongest correlation was between commitment and general satisfaction (r = 0.720), which aligns with theoretical expectations that committed individuals report higher relationship satisfaction. The moderate correlations between all constructs (r = 0.517–0.720) demonstrate convergent validity while remaining below 0.85, indicating that each scale captures distinct aspects of relationship quality rather than redundant constructs.

### 3.4. Criterion Validity

Criterion validity was assessed by correlating each scale score with the single-item relationship happiness measure at 6-month follow-up (n = 192). All scales demonstrated significant positive correlations with relationship happiness, confirming criterion validity. The commitment scale showed a strong positive correlation (r = 0.67, *p* < 0.001), as did the trust scale (r = 0.63, *p* < 0.001). Both satisfaction subscales demonstrated similarly strong associations, with the general satisfaction subscale correlating at r = 0.70 (*p* < 0.001) and the marital conflict/dissatisfaction subscale showing the strongest correlation at r = 0.72 (*p* < 0.001).

These strong correlations (r = 0.63–0.72) indicate that higher scores on each relationship quality domain are associated with greater overall relationship happiness, supporting the criterion validity of all validated scales.

### 3.5. Reliability Assessment

Internal Consistency: All scales demonstrated good to excellent internal consistency at both timepoints. Cronbach’s alpha values were: commitment scale α = 0.92 (baseline) and 0.96 (6-month); trust scale α = 0.90 (baseline) and 0.95 (6-month); general satisfaction subscale α = 0.83 (baseline) and 0.84 (6-month); and marital conflict/dissatisfaction subscale α = 0.79 (baseline) and 0.84 (6-month). All values exceeded the 0.70 threshold for acceptable reliability.

Test-Retest Reliability: Test-retest reliability over the 6-month period showed variable stability across scales: commitment scale r = 0.51 (*p* < 0.001, moderate reliability); trust scale r = 0.41 (*p* < 0.001, low-moderate reliability); marital conflict/dissatisfaction subscale r = 0.49 (*p* < 0.001, moderate reliability); and general satisfaction subscale r = 0.21 (*p* < 0.05, low reliability). The lower test-retest correlations may reflect genuine changes in relationship dynamics during the early stages of marriage rather than measurement instability.

## 4. Discussion

Our study successfully validated scales measuring trust, commitment, and satisfaction among newly married women in Nepal, addressing a critical gap in marital relationship quality measurement tools for South Asian populations. The validated measures, a 5-item commitment scale, 8-item trust scale, and 6-item satisfaction scale (comprising two subscales), demonstrated acceptable psychometric properties and provide researchers with reliable tools for relationship quality assessment in this cultural context.

All scales showed good internal consistency (α = 0.79–0.96) and significant criterion validity correlations with relationship happiness (r = 0.63–0.72). The commitment and trust scales replicated single-factor structures found in previous validation studies, including work in Ethiopia [27], suggesting these constructs may be relatively universal across cultures. The elevated RMSEA values for commitment (0.150) and trust (0.149) scales warrant careful consideration. While these values exceed the conventional RMSEA ≤ 0.08 threshold for good fit, several factors support the acceptability of our models. First, RMSEA is particularly sensitive to sample size, with smaller samples (n < 250) often producing inflated RMSEA values even when other fit indices indicate acceptable model fit [82,83]. Our CFA sample size of 192 falls within this range where RMSEA may be less reliable as a sole indicator of model adequacy. Second, the strong performance of other fit indices provides convergent evidence for model acceptability. Both scales demonstrated excellent CFI values (commitment: 0.983; trust: 0.954) and TLI values (commitment: 0.966; trust: 0.936) that exceed the 0.95 threshold, alongside acceptable SRMR values (commitment: 0.012; trust: 0.028). Given that our models met at least two of the four fit criteria and demonstrated strong factor loadings and reliability, we consider the single-factor structures acceptable for research use.

However, the satisfaction scale’s emergence as a two-factor structure, distinguishing marital conflict/dissatisfaction from general satisfaction, highlights important cultural nuances in how relationship satisfaction is conceptualized in the Nepali context. Notably, the marital conflict/dissatisfaction subscale showed the strongest association with happiness, suggesting that the absence of conflict and dissatisfaction is particularly important for overall relationship happiness.

The variable test-retest reliability over six months suggests the dynamic nature of newly formed marriages rather than measurement instability. In early marriage, particularly in arranged marriage contexts such as in Nepal, relationships undergo significant development as couples adjust to cohabitation with in-laws, negotiate roles, and build intimacy. The lower stability in trust and general satisfaction may capture genuine relationship evolution rather than psychometric weakness, supporting the scales’ sensitivity to meaningful change.

These validated scales enable crucial research linking marital quality to health outcomes in South Asian populations. Given meta-analytic evidence demonstrating associations between marital quality and physical health, mental wellbeing, and mortality in Western populations, validated measurement tools are essential for examining these relationships in non-Western contexts where marriage patterns, gender roles, and family dynamics differ substantially.

Our validation approach offers distinct advantages compared to previous relationship quality measurement efforts in South Asian contexts. Unlike studies that developed entirely new culture-specific instruments [14,59], our validation of established Western scales enables direct comparison with international research findings and facilitates meta-analytic studies. The brief nature of our validated scales (4–8 items each) provides practical advantages over longer instruments while maintaining acceptable reliability coefficients (α = 0.79–0.96). While custom-developed instruments may capture culture-specific relationship dynamics more comprehensively, our validated Western scales offer standardization that supports cross-cultural research comparisons. The emergence of a two-factor satisfaction structure in our Nepali sample, differing from typical Western unidimensional conceptualizations, suggests some cultural variation while maintaining psychometric acceptability. Future research combining our validated Western scales with qualitative exploration of culture-specific relationship dimensions could provide more comprehensive measurement approaches for South Asian populations.

### 4.1. Implications for Practice

The validated scales have several important practical applications beyond research settings. In clinical practice, these measures provide assessment tools for couples counselors and mental health professionals working with South Asian populations. The scales can support initial relationship assessment, treatment planning, and monitoring therapeutic progress over time. Healthcare providers, particularly those working in maternal and family health, could use these measures to screen for relationship distress during critical periods such as pregnancy and early parenthood, when relationship quality significantly impacts both maternal and child health outcomes.

The scales demonstrated reliability and validity also support their use in developing and evaluating couple-based interventions. Community health programs focused on family wellbeing could incorporate these measures to identify at-risk couples and target prevention efforts. For practitioners, we recommend computing scale scores as unweighted sums of retained items, with the satisfaction scale yielding two separate subscale scores. The relatively brief nature of these scales (4–8 items each) makes them feasible for routine clinical use while maintaining psychometric rigor.

### 4.2. Future Research Directions in Nepal

These validated measures enable several important research directions in Nepal. Future longitudinal studies could examine how relationship quality changes during major life transitions, such as the transition to parenthood or economic migration patterns common in Nepal. The measures could support research investigating relationships between marital quality and maternal mental health outcomes, particularly given Nepal’s challenges with maternal depression and anxiety. Additionally, these tools could facilitate research on how traditional arranged marriages evolve over time compared to self-choice marriages, an increasingly relevant question as marriage practices change in Nepal.

### 4.3. Strengths and Limitations

This study employed rigorous psychometric methodology using two waves of data for EFA and CFA. The community-based recruitment from rural Terai provided representative data for a key demographic (newly married women cohabiting with their in-laws) in Nepal, with excellent retention (96%) minimizing attrition bias. The comprehensive psychometric evaluation included factor structure, internal consistency, criterion validity, and test-retest reliability assessment. 

Several limitations warrant consideration. First, the focus on three positive relationship dimensions (trust, commitment, satisfaction) provides only partial coverage of relationship quality. While the satisfaction scale includes a conflict/dissatisfaction subscale, future validation should incorporate additional negative dimensions such as conflict resolution difficulties, communication problems, and relationship distress that may be particularly relevant in arranged marriage contexts. Second, sample homogeneity limits generalizability. Validation was limited to newly married women aged 18–25 in rural Terai, excluding male perspectives, longer-term marriages, and other demographic groups. Getting inputs from women only prevents examination of dyadic relationship dynamics. Cultural specificity to the Terai region may limit applicability to other South Asian populations, and validity for non-heterosexual relationships, different marriage arrangements, or non-marital partnerships remains unknown. Several scale items contained compound constructs (e.g., ‘honest and truthful’), which may have contributed to measurement complexity and could affect item interpretation [84]. Future scale development should consider simplifying item stems to focus on single constructs to improve clarity and potentially enhance psychometric properties. Third, we acknowledge that the 2-item general satisfaction subscale presents methodological limitations, as scales with fewer than three items have inherent reliability constraints. While this subscale demonstrated acceptable internal consistency (α = 0.83–0.84) and strong criterion validity (r = 0.70), future research should develop additional items to create a more robust positive satisfaction measure or consider focusing primarily on the 4-item conflict/dissatisfaction subscale, which may provide more stable psychometric properties. Fourth, our criterion validity assessment relied on a single-item relationship happiness measure, which limits the robustness of validity evidence compared to multi-item criterion measures. Single-item measures are more susceptible to measurement error and may not capture the full complexity of relationship satisfaction and happiness constructs. Finally, while our scales demonstrated acceptable psychometric properties, we acknowledge important limitations regarding cultural specificity. Our validation of Western-developed scales without cultural adaptation may not fully capture relationship dynamics unique to Nepali culture, such as the influence of extended family relationships or culture-specific expressions of trust and commitment. The emergence of a two-factor satisfaction structure may suggest some cultural variation, but this requires further investigation with qualitatively informed research.

Future validation studies should incorporate multi-item criterion measures, such as established relationship satisfaction scales, to provide more comprehensive validity evidence. Future research should consider expanding these scales to include comprehensive negative relationship dimensions, validate across diverse South Asian populations and relationship types, and examine predictive validity for health outcomes. From a public health perspective, these tools enable research on marital factors influencing maternal and child health, women’s mental health, and family health behaviors—crucial for developing evidence-based couple interventions in South Asian contexts.

## 5. Conclusions

This study provides psychometrically validated scales for measuring trust, commitment, and satisfaction among newly married women in Nepal, addressing a critical measurement gap in South Asian relationship research. The validated scales demonstrated acceptable reliability, validity, and model fit, establishing essential tools for investigating marriage-health associations in non-Western contexts.

These validated measures enable public health research examining how marital relationships influence maternal and child health outcomes, women’s mental health, and family health behaviors in South Asian populations. This measurement foundation supports the development of evidence-based, culturally appropriate interventions in low- and middle-income countries. Future research should expand validation to include negative relationship dimensions and test cross-cultural applicability across diverse South Asian populations and relationship types and collect dyadic data from both couple members.

## Figures and Tables

**Table 1:** Socio-demographic characteristics of newly married women in Nawalparsi district of Nepal at baseline (n = 200).

Variable	n (%) or Mean (SD)
Women’s age in years	20.4 (1.9)
Woman’s education	
No schooling	8 (4.0%)
Completed up to grade 5	24 (12.0%)
Completed grade 6 to 12	138 (69.0%)
Completed more than grade 12	30 (15.0%)
Religion	
Hindu	172 (86.0%)
Other	28 (14.0%)
Ethnicity/Caste	
Brahmin/Chhetri (Upper caste)	45 (22.5%)
Indigenous group	106 (53.0%)
Dalits/religious minority group (Lower caste)	49 (24.5%)
Marriage type	
Arranged	141 (70.5%)
Love	59 (29.5%)
Women’s paid work status outside home	52 (26.0%)
Husband’s age in years	23.8 (2.9)
Husband’s education	
No schooling	3 (1.5%)
Completed up to grade 5	19 (9.5%)
Completed grade 6 to 12	148 (74.4%)
Completed more than grade 12	29 (14.6%)

**Table 2:** Marital relationship quality scores by each item at baseline (n = 200).

	Marital Relationship Quality		
	Mean (M)	SD	Range
Commitment scale (5-items)	13.52	2.77	0–15
I expect my love for my current husband to last for the rest of my life (0 = not at all; 3 = extremely)	2.73	0.66	0–3
I can’t imagine ending my relationship with my current husband (0 = not at all; 3 = extremely)	2.72	0.65	0–3
I view my relationship with my current husband as permanent (0 = not at all; 3 = extremely)	2.74	0.61	0–3
I am committed to maintaining my relationship with my current husband (0 = not at all; 3 = extremely)	2.70	0.60	0–3
I have confidence in the stability of my relationship with my current husband (0 = not at all; 3 = extremely)	2.65	0.63	0–3
Trust scale (8-items)	18.10	4.14	0–24
My husband is primarily interested in his own welfare (0 = extremely; 3 = not at all) *	0.85	0.72	0–3
There are times when my husband cannot be trusted (0 = extremely; 3 = not at all) *	1.00	0.78	0–3
My husband is perfectly honest and truthful with me (0 = strongly disagree; 3 = strongly agree)	2.34	0.65	0–3
I feel I can trust my husband completely (0 = strongly disagree; 3 = strongly agree)	2.35	0.62	0–3
My husband is truly sincere in his promises (0 = strongly disagree; 3 = strongly agree)	2.32	0.65	0–3
I feel that my husband does not show me enough consideration (0 = extremely; 3 = not at all)*	0.78	0.68	0–3
My husband treats me fairly and justly (0 = strongly disagree; 3 = strongly agree)	2.32	0.64	0–3
I feel that my husband can be counted on to help me (0 = strongly disagree; 3 = strongly agree)	2.39	0.63	0–3
Satisfaction scale (7-items)	18.50	2.65	3–21
How often have you discussed, or have you considered divorce separation or termination in your relationship? (0 = all of the time; 3 = never) *	2.95	0.12	1–3
How often do you or your husband leave the house after a fight? (0 = all of the time; 3 = never) *	2.99	0.12	2–3
In general, how often do you think that things between you and your husband are going well? (0 = never; 3 = all the time)	2.39	0.82	0–3
Do you confide in your husband?	2.39	0.78	0–3
Do you ever regret that you are married? (0 = all of the time; 3 = never) *	2.85	0.54	0–3
How often do you and your husband quarrel? (0 = all of the time; 3 = never) *	2.87	0.48	0–3
How often do you and your husband “get on each other’s nerves?” (0 = all of the time; 3 = never) *	2.09	0.67	0–3

Note: * indicates reverse-coded items. All scales: higher scores = better relationship quality.

**Table 3:** Factor loadings from exploratory factor analysis for items for commitment, trust, and satisfaction scales at baseline (n = 200).

	Factor Loadings		Communality (h^2^)
Commitment scale (5-items; 1 factor)			
I expect my love for my current husband to last for the rest of my life	0.76		0.584
I can’t imagine ending my relationship with my current husband	0.87		0.759
I view my relationship with my current husband as permanent	0.90		0.813
I am committed to maintaining my relationship with my current husband	0.87		0.763
I have confidence in the stability of my relationship with my current husband	0.85		0.722
Trust scale (8-items; 1 factor)			
My husband is primarily interested in his own welfare	0.57		0.329
There are times when my husband cannot be trusted	0.52		0.272
My husband is perfectly honest and truthful with me	0.68		0.471
I feel I can trust my husband completely	0.86		0.742
My husband is truly sincere in his promises	0.87		0.762
I feel that my husband does not show me enough consideration	0.62		0.396
My husband treats me fairly and justly	0.90		0.828
I feel that my husband can be counted on to help me	0.89		0.792
Satisfaction scale (7-items)	Factor 1	Factor 2	Communality (h^2^)
Factor 1: Marital Conflict/Dissatisfaction (4 items)			
How often have you discussed divorce/separation? (R)	0.78	0.04	0.645
How often do you/husband leave house after a fight? (R)	0.75	0.01	0.567
Do you ever regret that you are married? (R)	0.78	0.00	0.605
How often do you and your husband quarrel? (R)	0.81	−0.05	0.622
Factor 2: General Satisfaction (2 items)			
How often do you think that things between you and your husband are going well?	−0.01	0.78	0.609
Do you confide in your husband?	−0.00	0.82	0.672
Item dropped due to poor loading			
How often do you and your husband “get on each other’s nerves?” (R)	0.27	0.34	0.280
**EFA Summary Statistics**			
Scale	Eigenvalue	Variance	Cumulative %
Commitment (1 factor)	3.64	72.8%	72.8%
Trust (1 factor)	4.59	57.4%	57.4%
Satisfaction Factor 1	3.17	45.2%	45.2%
Satisfaction Factor 2	0.83	11.9%	57.1%

Note: (R) = reverse-coded items. Oblique rotation used for satisfaction scale.

**Table 4:** Standardized factor loadings from confirmatory factor analysis using the second wave of data (n = 192).

	Standardized Factor Loadings	
Commitment scale (5-items)		
I expect my love for my current husband to last for the rest of my life	0.94	
I can’t imagine ending my relationship with my current husband	0.92	
I view my relationship with my current husband as permanent	0.92	
I am committed to maintaining my relationship with my current husband	0.92	
I have confidence in the stability of my relationship with my current husband	0.94	
Trust scale (8-items)		
My husband is primarily interested in his own welfare (R)	0.71	
There are times when my husband cannot be trusted (R)	0.68	
My husband is perfectly honest and truthful with me	0.94	
I feel I can trust my husband completely	0.95	
My husband is truly sincere in his promises	0.94	
I feel that my husband does not show me enough consideration(R)	0.82	
My husband treats me fairly and justly	0.95	
I feel that my husband can be counted on to help me	0.86	
Satisfaction scale (7-items)	Factor 1	Factor 2
Factor 1: Marital Conflict/Dissatisfaction (4 items)		
How often have you discussed divorce/separation? (R)	0.86	-
How often do you/husband leave house after a fight? (R)	0.75	-
Do you ever regret that you are married? (R)	0.84	-
How often do you and your husband quarrel? (R)	0.74	-
Factor 2: General Satisfaction (2 items)		
How often do you think that things between you and your husband are going well?	-	0.80
Do you confide in your husband?	-	0.91

Note: (R) = reverse-coded items. Dashes (-) indicate items not loading on that factor. One item (“get on each other’s nerves”) was dropped after EFA. Dashes (-) indicate items not loading on that factor.

**Table 5:** Model fit statistics from confirmatory factor analysis (CFA) at 6-month follow-up data (n = 192).

Factor Structure	RMSEA	CFI	TLI	SRMR
Commitment scale (1 factor)	0.150	0.983	0.966	0.012
Trust scale (1 factor)	0.149	0.954	0.936	0.028
Satisfaction scale (1 factor)	0.257	0.794	0.691	0.084
Satisfaction scale (2 factors)	0.088	0.981	0.965	0.035

Note: Good model fit indicated by: RMSEA ≤ 0.08 (adequate fit ≤ 0.10), CFI ≥ 0.95, TLI ≥ 0.95, SRMR ≤ 0.10. Models meeting at least two criteria considered acceptable fit.

**Table 6:** Correlation analysis of scales to measure marital relationship quality using follow-up wave 2 data.

	Commitment	Trust	General Satisfaction	Marital Conflict and Dissatisfaction
**Commitment**	1.000	-	-	-
**Trust**	0.613	1.000	-	-
**General Satisfaction**	0.720	0.595	1.000	-
**Marital conflict and dissatisfaction**	0.661	0.517	0.639	1.000

Note: All correlations significant at *p* < 0.05 level.

## Data Availability

Data are available upon reasonable request. The data that support the findings of this study are available from the corresponding author, LG, upon reasonable request and with appropriate ethical permissions.
